# Cross-Sectional Association Between Types of Leisure Activities and Self-rated Health According to Gender and Work Status Among Older Japanese Adults

**DOI:** 10.2188/jea.JE20180108

**Published:** 2019-11-05

**Authors:** Kimiko Tomioka, Norio Kurumatani, Keigo Saeki

**Affiliations:** Nara Prefectural Health Research Center, Nara Medical University, Nara, Japan

**Keywords:** gender difference, leisure activities, self-rated health, successful aging, work status

## Abstract

**Background:**

Participation in leisure activities (LA) is essential for successful aging. Our aim was to investigate the cross-sectional association of types of LA with self-rated health (SRH) by gender and work status.

**Methods:**

The target population was all residents aged ≥65 years in a municipality (*n* = 16,010; response rate, 62.5%). We analyzed 4,044 men and 4,617 women without disabilities. LA were categorized into 14 types. SRH was assessed through the SF-8. Excellent or very good SRH was defined as positive SRH. Covariates included age, marital status, education, subjective economic status, body mass index, chronic diseases, alcohol, smoking, walking time, depression, and cognitive functioning. Multiple logistic regressions were used to calculate the odds ratio (OR) and 95% confidence interval (CI) for positive SRH, with non-participation as the reference.

**Results:**

After adjustment for covariates and mutual adjustment for other LA, participation in the following types of LA was positively associated with positive SRH: sports activities among working men (OR 1.46; 95% CI, 1.07–2.00), non-working men (OR 1.33; 95% CI, 1.04–1.69), and non-working women (OR 1.74; 95% CI, 1.41–2.15); cooking among non-working men (OR 1.65; 95% CI, 1.18–2.33) and non-working women (OR 1.28; 95% CI, 1.03–1.60); musical activities among working men (OR 1.44; 95% CI, 1.01–2.05) and non-working women (OR 1.59; 95% CI, 1.29–1.95); and technology usage only among working men (OR 1.41; 95% CI, 1.01–1.96). In contrast, TV watching was negatively associated with positive SRH among non-working women (OR 0.69; 95% CI, 0.56–0.85).

**Conclusions:**

Our results suggest that encouraging older adults to participate in types of LA appropriate to their gender and work status might be a key to positive SRH.

## INTRODUCTION

In our rapidly aging society, health priorities for older people are not just about extending lives, but extending years of being active; in other words, maintaining independent living. One key contributor to successful aging is said to be older people’s engagement in leisure activities (LA).^1^

LA later in life are associated with a reduced risk of adverse health outcomes, such as functional decline,^2^ cognitive decline,^3^ and decreased quality of life.^4^ However, previous studies lack a consistent definition of LA, and there are few that report on the health effects of different types of LA. There are various types of LA, including physical activities, such as sports, which mainly use body function, and mental activities, such as cultural activities, which do not require much physical functioning but require high-level mental functioning. Therefore, it is possible that LA affect aged people in different ways depending on their type.^5^ Additionally, it has been pointed out in a prior study that men and women often preferred different activities.^6^ However, there are a limited number of studies concerning gender differences in regards to health effects of different types of LA: Nilsen et al reported that solitary activities, such as solving crossword puzzles, predicted longer survival only for women^5^; in a study by Agahi and Parker, solitary activities, such as hobby activities and gardening, were associated with a lower mortality risk only in men^7^; Takeda et al found that gardening could prevent progressive dementia-associated senility in men, while sports activities were beneficial only for older women^8^; and Gautam et al reported that TV watching correlated to lower depression for both genders, while physical exercise related to lower depression only for older men.^9^ Although the results of prior studies cannot be compared because of their different LA definitions and outcomes, they suggest that it is vital to conduct stratified analyses by gender.

Older adults used to withdraw from the labor population at retirement age. Amid the decline in the working-age population, workforce participation by older adults has been encouraged with a view towards addressing labor shortages and reducing the social security burden. Although we have no sufficient evidence on health impact by extending working life, some prior studies suggest that working in later life may provide older adults with opportunities for social integration and a stronger sense of belonging and purpose, leading to health maintenance.^10^^,^^11^ Additionally, there are several reports that the LA-health relationship among older people may be affected by work status. For example, Sugihara et al examined the relationship of productive activities, such as paid work, unpaid work at home, and volunteering, with depressive symptoms among late-middle-aged Japanese adults.^12^ They found that the non-working population tended to have more depressive symptoms compared to the working population, but retired men engaged in volunteer activities had no increase in depressive symptoms. Lee et al investigated the association between leisure activity engagement, retirement, and cognition among older Americans.^13^ They reported that continuously non-working people showed significantly lower cognition than those who were continuously working, but retired individuals involved in mental activities realized a protective effect on cognitive decline. A meta-analysis conducted by Kuykendall et al has reported that retired persons show a stronger relationship between leisure engagement and subjective well-being than working people.^14^ These studies^12^^–^^14^ suggest that LA in later life may provide alternatives to work for retired older adults and attenuate the negative effects of not working. Therefore, it is possible that non-working older adults may receive more health benefits from LA compared to the working population, and that the association between LA and the health of older people may depend not only on gender but also on working status.

Our study used self-rated health (SRH) as a health indicator. The reasons are: 1) SRH is used globally as a health index that can easily collect information related to qualitative aspects of health, which objective health indices, such as mortality and morbidity, cannot pick up,^15^^,^^16^ and 2) previous research has demonstrated that the SRH of older people was not only an independent predictor of instrumental activities of daily living (IADL) change, but also showed a significant dose-response relationship between better SRH and more IADL maintenance.^17^ Therefore, by using SRH as a health indicator, it is possible to identify LA that maintain IADL and contribute positively to healthy longevity in older people.

This study sought to examine the cross-sectional relationship between SRH and LA from the perspective of the type of LA among community-dwelling older adults, and to verify whether the association between SRH and LA may vary across gender and work status.

## METHODS

### Study participants

In November 2016, self-administered questionnaires were mailed to all individuals 65 years of age or more living in A City in Nara Prefecture, Japan (*n* = 16,010). As of November 30, 2016, the targeted municipality had a population of 78,987 and an aging population rate of 21.9%. Because physical functioning strongly affects both SRH and daily activities,^15^^,^^17^^,^^18^ participants eligible for inclusion in this study were limited to individuals who had no disability in basic activities of daily living (BADL). Individuals with BADL disability were defined as those who were certified by the municipal government as being in need of care, or those who reported needing help to perform any of the five BADL items: eating, dressing, bathing, going to the toilet, and walking indoors.

Information regarding care need assessments conducted by municipal officers, age, and gender was provided by A City, and other data were obtained from the self-administered questionnaires. Ethical approval for the study protocol was obtained from the Nara Medical University Ethics Committee (approval number 939). Submission of the self-administered questionnaires was considered to be consent for participating in the research.

### Measurements

#### Assessment of leisure activities (LA)

We classified LA into 14 types (eTable 1): sports activities, gardening, musical activities, creative activities, cultural activities, playing games, sightseeing, art appreciation, TV watching, cooking, pet ownership, technology usage, investment, and gambling. LA were measured through the question “Currently, do you participate in the following LA?” Subjects could answer “currently participate” or “do not participate” for each type of LA. Although the main aim of this study was to evaluate the association between each type of LA and SRH, the total number of types of LA in which each subject engaged was also calculated as an indicator of overall LA participation. The number of types of LA was handled as a continuous variable.

Sports activities and gardening are physical activities that community-dwelling older adults engage in frequently.^6^^,^^8^^,^^9^^,^^19^^–^^21^ Musical activities, creative activities, cultural activities, and playing games are classified as mental activities or cognitive activities^3^^,^^22^ and have been examined for their effects on cognitive functioning. Aartsen et al defined sightseeing and art appreciation as experiential activities, and investigated their relation to cognitive performance.^23^ Some researchers consider TV watching,^24^ cooking,^25^ and pet ownership^26^ as one type of LA in older adults, and have studied their impact on health.

Moreover, great changes in modern lifestyle possibly have an impact on the LA of today’s aged population. For example, 1) the spread of information and communication technologies has created a new lifestyle based around the Internet, e-mail, and mobile/smart phones,^27^ and 2) previous researchers combined investment and gambling into one type of LA.^8^ However, with recent changes to pensions and within the financial system generally, older people’s investments have become a common and important measure of asset management. Therefore, in this study, technology usage was included as one type of LA, and investment and gambling were included as separate types of LA.

#### Assessment of self-rated health (SRH)

We evaluated SRH using one item (that is, general health) from the Japanese version of the 8-item Short-Form Health Survey (SF-8).^28^ The SF-8 has adequate validity and reliability,^28^^,^^29^ and consists of the following eight subscales: physical functioning, role-physical, bodily pain, general health, vitality, social functioning, role-emotional, and mental health. For general health, the question is: “Overall, how would you rate your health during the past four weeks? Was it excellent, very good, good, fair, poor, or very poor?”. Based on this item, individuals who rated their general health excellent or very good were defined as those with positive SRH.

#### Work status

Work status was measured using the question “Currently, are you in paid employment? Yes or no?”, and categorized into subjects with or without paid work.

#### Covariates

Based on prior studies,^2^^,^^4^^–^^7^ the following variables were adopted as covariates which may correlate with LA and SRH: age, socioeconomic status (marital status, education, and subjective economic status), health status (body mass index [BMI] and self-reported chronic diseases), and health behaviors (alcohol, smoking, and walking time). Additionally, there may be a link between mental function, specifically depression^9^^,^^17^ and cognitive functioning,^3^^,^^8^ LA, and SRH, and different mechanisms may be involved depending on the type of LA. Therefore, we added depression and cognitive functioning to the model adjusted for covariates. In line with the statistical recommendation relative to missing covariates,^30^ we performed multiple imputations by means of chained equations.

Details of the covariates and multiple imputations are described in the supplementary data (eAppendix 1), and information including the number of missing values according to gender and work status is shown in eTable 2.

### Statistical analysis

Multiple logistic regression was used to assess the relationship between LA and SRH. An explanatory variable was LA (both the type of LA and the number of the types of LA), with non-participation as the reference group. We calculated the odds ratio (OR) for positive SRH and a 95% confidence interval (CI). The analyses were classified according to work status and gender group: working men, non-working men, working women, and non-working women. Model 1 showed the calculations of crude OR for positive SRH. Model 2 was adjusted for covariates. In model 3, depression and cognitive functioning were added to the variables in model 2. To evaluate the independent association between each type of LA and positive SRH in model 4, we conducted mutual adjustment for all 14 types of LA, in addition to adjustment for covariates and mental function. We also examined the interaction between work status and each type of LA and the interaction between gender and each type of LA, in relation to positive SRH.

All analyses were performed using IBM SPSS Statistics version 24.0 (IBM Corporation, Armonk, NY, USA), with a significance level of 5%.

## RESULTS

A total of 10,009 persons responded (response rate, 62.5%). The proportion of individuals who were identified by the municipal government as being in need of care was 5.6% in respondents and 11.1% in non-respondents, showing that non-respondents had a significantly poorer BADL than respondents (chi-square test, *P* < 0.001). Among individuals who were identified as not needing care at the time of the survey, non-respondents were significantly younger than respondents, but were comparable in terms of gender (Table 1). Among the 9,445 respondents not needing care, 784 persons were excluded from analysis because of BADL dependence based on the questionnaire (*n* = 155) or missing data for LA, SRH, and/or work status (*n* = 629). Finally, 8,661 persons (4,044 men and 4,617 women) were included in this study.

**Table 1.** Basic attributes of respondents and non-respondents to the questionnaire^a^

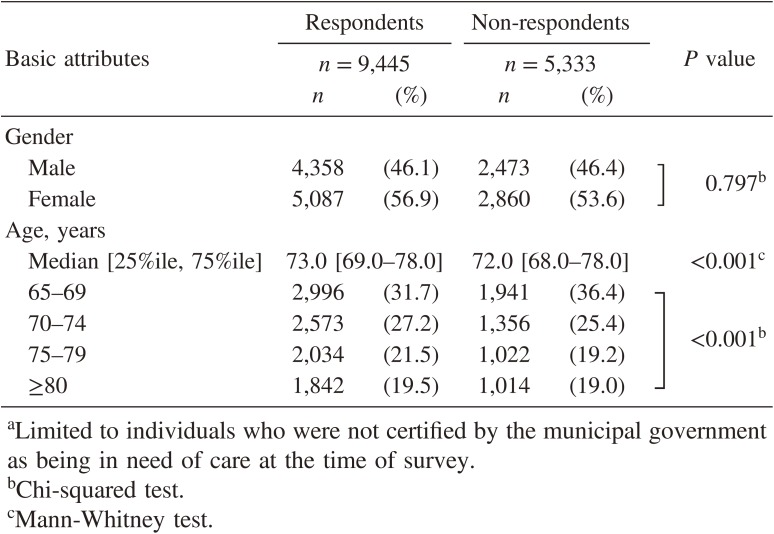

^a^Limited to individuals who were not certified by the municipal government as being in need of care at the time of survey.

^b^Chi-squared test.

^c^Mann-Whitney test.

### General characteristics of the participants

Characteristics of study participants are shown in Table 2. The mean age was 73.6 (standard deviation [SD], 6.0) in men and 73.6 (SD, 6.4) in women, showing no gender difference (*P* = 0.916). The proportion of participants with positive SRH was 20.3% for men and 16.3% for women, showing a significant gender difference (chi-square test, *P* < 0.001). Men were more likely to be working and to participate in sports activities, playing games, sightseeing, watching TV, technology usage, investment, and gambling. In contrast, women were more likely to participate in musical activities, creative activities, cultural activities, art appreciation, and cooking. Working persons were more likely to be younger, have positive SRH, and participate in sightseeing, pet ownership, technology usage, investment, and gambling. In contrast, non-working persons tended to participate in creative activities, cultural activities, and cooking.

**Table 2.** Characteristics of study participants according to gender and work status

	Men (*n* = 4,044)		Women (*n* = 4,617)	
	
	Working (*n* = 1,208)	Not working (*n* = 2,836)	Working (*n* = 687)	Not working (*n* = 3,930)
Age, years, mean (SD)	71.0 (4.9)	74.8 (6.1)	70.1 (4.5)	74.3 (6.5)
Socio-economic status				
Marital status: married	91.5%	87.9%	64.5%	62.6%
Education: junior college or higher	39.8%	29.4%	27.1%	19.5%
Subjective economic status: rich	30.9%	20.8%	28.2%	23.5%
Health status				
Body mass index, kg/m^2^, mean (SD)	23.3 (2.8)	23.1 (3.6)	22.5 (3.1)	22.6 (4.2)
Chronic diseases: none	35.9%	30.8%	48.2%	39.2%
Health behaviors				
Alcohol: daily/occasional drinkers	68.8%	60.3%	36.1%	26.6%
Smoking: ex/current smokers	73.3%	73.1%	13.2%	8.8%
Walking time: ≥1 hour per day	39.8%	31.9%	39.7%	31.2%
Mental function				
Depression: present	12.9%	27.1%	18.3%	27.4%
Cognitive functioning: poor	12.7%	21.4%	7.4%	16.5%
Self-rated health				
Subjects with positive SRH	24.3%	18.6%	24.5%	14.8%
Leisure activity participation				
Sports activities	56.3%	60.5%	45.1%	46.9%
Gardening	41.1%	47.7%	47.3%	44.5%
Musical activities	20.0%	20.9%	27.1%	27.2%
Creative activities	12.3%	15.5%	25.6%	26.2%
Cultural activities	18.6%	22.1%	32.0%	30.3%
Playing games	19.4%	22.7%	2.6%	4.0%
Sightseeing	52.5%	47.4%	49.8%	45.2%
Art appreciation	29.0%	29.5%	38.3%	37.7%
TV watching	58.4%	60.1%	55.9%	53.0%
Cooking	7.3%	7.7%	27.7%	26.0%
Pet ownership	16.6%	13.2%	15.4%	12.4%
Technology usage	39.8%	34.9%	24.6%	16.5%
Investment	18.4%	17.5%	10.3%	7.7%
Gambling	20.4%	19.1%	6.4%	4.3%
Number of leisure activities, mean (SD)	4.2 (2.5)	4.3 (2.6)	4.2 (2.6)	3.9 (2.7)

SD, standard deviation; SRH, self-rated health.

### Cross-sectional relationship between LA and positive SRH

Crude and adjusted odds ratios for positive SRH associated with types of LA by work status are reported in Table 3 for men and Table 4 for women. In the crude model (model 1), regardless of gender or work status, sport activities, cultural activities, sightseeing, art appreciation, and technology usage were each associated with positive SRH, while there was no significant association between playing games and positive SRH. Other types of LA had significant associations with positive SRH, according to gender and work status. After adjusting for covariates (model 2), many types of LA became non-significant, but the following types of LA remained significant: regardless of work status, sport activities and technology usage in men, and gardening, sightseeing, art appreciation, and cooking in women; among working men, musical activities and art appreciation; among non-working men, creative activities, sightseeing, and cooking; and among non-working women, sport activities, musical activities, and technology usage. After additional adjustment for depression and cognitive functioning (model 3), the ORs were attenuated but remained significant in the following types of LA: regardless of work status, sport activities in men, and sightseeing and cooking in women; among working men, musical activities, art appreciation, and technology usage; among non-working men, sightseeing and cooking; among working women, gardening and art appreciation; and among non-working women, sports activities and musical activities. In the final model (model 4), where the data were mutually adjusted for all types of LA, the adjusted ORs for types of LA that were statistically significant in model 3 tended towards 1.00, but significant differences between participation and non-participation were observed in some types of LA, depending on gender and work status. Sports activities had a significantly higher OR for positive SRH in working men (OR 1.46; 95% CI, 1.07–2.00), non-working men (OR 1.33; 95% CI, 1.04–1.69), and non-working women (OR 1.74; 95% CI, 1.41–2.15). Cooking was positively associated with positive SRH in non-working men (OR 1.65; 95% CI, 1.18–2.33) and non-working women (OR 1.28; 95% CI, 1.03–1.60). Musical activities showed a positive association with positive SRH in working men (OR 1.44; 95% CI, 1.01–2.05) and non-working women (OR 1.59; 95% CI, 1.29–1.95). Moreover, the interaction between musical activities and work status was significant among men (*P* for interaction = 0.046; eTable 3), suggesting that working men were more likely to benefit from participation in musical activities compared to non-working men. Regarding technology usage, only working men had a positive association with positive SRH (OR 1.41; 95% CI, 1.01–1.96). Among non-working women, TV watching was significantly associated with a lower prevalence of positive SRH (OR 0.69; 95% CI, 0.56–0.85). Furthermore, there was a significant interaction between TV watching and work status among women (*P* for interaction = 0.043; eTable 4), demonstrating that TV watching was less beneficial in non-working women than in working women.

**Table 3.** Crude and adjusted odds ratios for positive self-rated health associated with the type of leisure activities by work status in 4,044 men

Type of LA	Model 1: Crude OR		Model 2^a^: Adjusted for covariates		Model 3^b^: Model 2 plus mental function		Model 4^c^: Model 3 plus mutual adjustment for all LA	
			
	Working *n* = 1,208 OR (95% CI)	Not working *n* = 2,836 OR (95% CI)	Working *n* = 1,208 OR (95% CI)	Not working *n* = 2,836 OR (95% CI)	Working *n* = 1,208 OR (95% CI)	Not working *n* = 2,836 OR (95% CI)	Working *n* = 1,208 OR (95% CI)	Not working *n* = 2,836 OR (95% CI)
Sports activities	**1.84 (1.39–2.42)**	**2.05 (1.66–2.52)**	**1.60 (1.19–2.14)**	**1.45 (1.16–1.83)**	**1.54 (1.15–2.07)**	**1.34 (1.06–1.69)**	**1.46 (1.07–2.00)**	**1.33 (1.04–1.69)**
Gardening	1.11 (0.85–1.45)	**1.33 (1.10–1.60)**	1.10 (0.83–1.45)	1.16 (0.95–1.41)	1.06 (0.80–1.40)	1.04 (0.85–1.28)	0.97 (0.73–1.31)	0.99 (0.80–1.22)
Musical activities	**1.72 (1.26–2.34)**	1.16 (0.92–1.45)	**1.69 (1.22–2.35)**	1.10 (0.87–1.40)	**1.60 (1.15–2.22)**	1.02 (0.80–1.30)	**1.44 (1.01–2.05)**	0.96 (0.74–1.24)
Creative activities	**1.54 (1.06–2.24)**	**1.51 (1.18–1.92)**	1.44 (0.97–2.15)	**1.38 (1.07–1.78)**	1.45 (0.97–2.17)	1.27 (0.98–1.64)	1.19 (0.77–1.83)	1.20 (0.91–1.58)
Cultural activities	**1.54 (1.12–2.12)**	**1.27 (1.02–1.58)**	1.38 (0.98–1.95)	1.06 (0.83–1.34)	1.38 (0.97–1.96)	0.97 (0.77–1.24)	1.09 (0.74–1.60)	0.88 (0.68–1.14)
Playing games	1.26 (0.91–1.74)	0.98 (0.78–1.22)	1.15 (0.82–1.61)	0.91 (0.72–1.16)	1.14 (0.81–1.60)	0.87 (0.68–1.11)	1.01 (0.70–1.45)	0.85 (0.66–1.09)
Sightseeing	**1.32 (1.01–1.72)**	**1.77 (1.46–2.15)**	1.27 (0.96–1.68)	**1.43 (1.16–1.76)**	1.21 (0.91–1.61)	**1.24 (1.00–1.53)**	1.00 (0.72–1.39)	1.18 (0.93–1.50)
Art appreciation	**1.56 (1.18–2.06)**	**1.45 (1.19–1.77)**	**1.45 (1.07–1.96)**	1.24 (1.00–1.53)	**1.39 (1.02–1.88)**	1.09 (0.88–1.35)	1.20 (0.85–1.71)	0.99 (0.77–1.27)
TV watching	1.10 (0.84–1.44)	**1.23 (1.01–1.49)**	1.04 (0.79–1.38)	1.07 (0.87–1.32)	0.99 (0.74–1.31)	0.95 (0.77–1.18)	0.82 (0.59–1.13)	0.87 (0.69–1.10)
Cooking	1.11 (0.68–1.83)	**1.85 (1.35–2.52)**	1.14 (0.68–1.92)	**1.88 (1.35–2.62)**	1.11 (0.66–1.86)	**1.69 (1.21–2.36)**	0.89 (0.52–1.54)	**1.65 (1.18–2.33)**
Pet ownership	1.01 (0.71–1.43)	0.99 (0.75–1.31)	0.94 (0.65–1.36)	0.95 (0.71–1.27)	0.93 (0.64–1.35)	0.90 (0.67–1.21)	0.85 (0.58–1.27)	0.87 (0.64–1.18)
Technology usage	**1.60 (1.23–2.08)**	**1.62 (1.33–1.96)**	**1.52 (1.13–2.04)**	**1.27 (1.02–1.57)**	**1.49 (1.11–2.00)**	1.14 (0.92–1.42)	**1.41 (1.01–1.96)**	1.08 (0.86–1.37)
Investment	1.30 (0.94–1.81)	**1.55 (1.23–1.95)**	1.03 (0.72–1.47)	1.13 (0.88–1.45)	1.02 (0.71–1.46)	1.05 (0.81–1.35)	0.88 (0.61–1.29)	1.00 (0.77–1.31)
Gambling	0.77 (0.55–1.09)	**0.78 (0.60–1.00)**	0.81 (0.57–1.16)	0.82 (0.63–1.07)	0.82 (0.57–1.18)	0.86 (0.66–1.13)	0.79 (0.54–1.15)	0.86 (0.65–1.13)

Overall activity^d^	**1.12 (1.06–1.18)**	**1.12 (1.08–1.16)**	**1.09 (1.03–1.16)**	**1.06 (1.02–1.10)**	**1.08 (1.02–1.15)**	1.03 (0.98–1.07)	—	—

CI, confidence interval; LA, leisure activities; OR, odds ratio.

Non-participation in each activity was set as the reference group. Results in bold indicate *P* < 0.05.

^a^Adjusted for age, marital status, education, subjective economic status, body mass index, chronic diseases, alcohol, smoking, and walking time.

^b^In addition to Model 2, depression and cognitive functioning were included.

^c^In addition to Model 3, all 14 types of LA were included (ie, Model 4 was mutually adjusted for all LA).

^d^Overall activity was measured by the number of leisure activities (per 1 increase).

**Table 4.** Crude and adjusted odds ratios for positive self-rated health associated with the type of leisure activities by work status in 4,617 women

Type of LA	Model 1: Crude OR		Model 2^a^: Adjusted for covariates		Model 3^b^: Model 2 plus mental function		Model 4^c^: Model 3 plus mutual adjustment for all LA	
			
	Working *n* = 687 OR (95% CI)	Not working *n* = 3,930 OR (95% CI)	Working *n* = 687 OR (95% CI)	Not working *n* = 3,930 OR (95% CI)	Working *n* = 687 OR (95% CI)	Not working *n* = 3,930 OR (95% CI)	Working *n* = 687 OR (95% CI)	Not working *n* = 3,930 OR (95% CI)
Sports activities	**1.78 (1.25–2.53)**	**2.41 (2.00–2.89)**	1.45 (0.99–2.11)	**2.01 (1.65–2.44)**	1.38 (0.94–2.02)	**1.81 (1.49–2.21)**	1.19 (0.79–1.78)	**1.74 (1.41–2.15)**
Gardening	**1.58 (1.12–2.25)**	**1.44 (1.21–1.72)**	**1.62 (1.12–2.35)**	**1.24 (1.03–1.49)**	**1.50 (1.03–2.20)**	1.13 (0.94–1.37)	1.32 (0.86–2.01)	1.06 (0.87–1.29)
Musical activities	1.39 (0.95–2.03)	**1.95 (1.63–2.35)**	1.38 (0.92–2.07)	**1.81 (1.50–2.20)**	1.28 (0.85–1.93)	**1.71 (1.41–2.08)**	1.07 (0.69–1.66)	**1.59 (1.29–1.95)**
Creative activities	1.22 (0.83–1.80)	**1.29 (1.06–1.56)**	1.17 (0.77–1.76)	1.14 (0.94–1.40)	1.10 (0.72–1.67)	1.03 (0.84–1.26)	0.82 (0.52–1.30)	0.92 (0.74–1.15)
Cultural activities	**1.59 (1.11–2.28)**	**1.32 (1.10–1.59)**	1.33 (0.89–1.97)	1.16 (0.95–1.42)	1.23 (0.83–1.84)	1.07 (0.87–1.30)	0.98 (0.62–1.53)	0.93 (0.75–1.16)
Playing games	1.57 (0.58–4.24)	1.30 (0.86–1.96)	2.45 (0.81–7.39)	1.16 (0.75–1.79)	2.65 (0.85–8.32)	0.99 (0.64–1.54)	2.10 (0.67–6.62)	0.82 (0.53–1.28)
Sightseeing	**1.80 (1.26–2.56)**	**1.74 (1.46–2.08)**	**1.66 (1.13–2.44)**	**1.37 (1.13–1.65)**	**1.51 (1.02–2.23)**	**1.22 (1.01–1.48)**	1.19 (0.75–1.89)	1.03 (0.83–1.29)
Art appreciation	**1.72 (1.21–2.45)**	**1.57 (1.32–1.88)**	**1.57 (1.08–2.29)**	**1.34 (1.11–1.61)**	**1.49 (1.01–2.18)**	1.17 (0.97–1.41)	1.22 (0.78–1.92)	1.03 (0.82–1.29)
TV watching	**1.43 (1.00–2.05)**	1.02 (0.85–1.21)	1.39 (0.96–2.03)	0.94 (0.78–1.13)	1.31 (0.90–1.93)	0.88 (0.73–1.06)	1.10 (0.72–1.67)	**0.69 (0.56–0.85)**
Cooking	**1.85 (1.28–2.68)**	**1.78 (1.47–2.14)**	**1.64 (1.10–2.43)**	**1.51 (1.24–1.83)**	**1.51 (1.01–2.27)**	**1.32 (1.08–1.61)**	1.37 (0.89–2.12)	**1.28 (1.03–1.60)**
Pet ownership	0.83 (0.51–1.37)	**1.29 (1.00–1.66)**	0.82 (0.49–1.39)	1.18 (0.91–1.53)	0.78 (0.46–1.33)	1.13 (0.87–1.47)	0.68 (0.39–1.18)	1.10 (0.84–1.45)
Technology usage	**1.48 (1.01–2.18)**	**1.60 (1.29–1.99)**	1.19 (0.78–1.80)	**1.33 (1.06–1.68)**	1.19 (0.78–1.81)	1.22 (0.97–1.55)	0.95 (0.60–1.50)	1.13 (0.88–1.44)
Investment	**1.93 (1.15–3.24)**	**1.70 (1.28–2.27)**	1.52 (0.86–2.70)	1.23 (0.91–1.67)	1.51 (0.85–2.71)	1.16 (0.85–1.57)	1.33 (0.73–2.42)	1.07 (0.78–1.47)
Gambling	1.03 (0.51–2.09)	0.76 (0.47–1.23)	1.23 (0.58–2.60)	0.84 (0.51–1.37)	1.10 (0.52–2.35)	0.81 (0.49–1.32)	1.01 (0.46–2.19)	0.70 (0.42–1.16)

Overall activity^d^	**1.16 (1.08–1.24)**	**1.15 (1.11–1.19)**	**1.13 (1.05–1.21)**	**1.11 (1.07–1.15)**	**1.11 (1.03–1.19)**	**1.07 (1.03–1.11)**	—	—

CI, confidence interval; LA, leisure activities; OR, odds ratio.

Non-participation in each activity was set as reference. Results in bold indicate *P* < 0.05.

^a^Adjusted for age, marital status, education, subjective economic status, body mass index, chronic diseases, alcohol, smoking, and walking time.

^b^In addition to Model 2, depression and cognitive functioning were included.

^c^In addition to Model 3, all 14 types of LA were included (ie, Model 4 was mutually adjusted for all LA).

^d^Overall activity was measured by the number of leisure activities (per 1 increase).

Regarding the number of types of LA, after adjusting for all covariates, an increased number of LA was significantly associated with higher prevalence of persons with positive SRH in working men (OR 1.08; 95% CI, 1.02–1.15), working women (OR 1.11; 95% CI, 1.03–1.19), and non-working women (OR 1.07; 95% CI, 1.03–1.11).

Regarding the interaction between gender and each type of LA (eTable 5), a significant interaction effect by gender was observed in musical activities (*P* for interaction = 0.022), showing that women were more likely to benefit from musical activities than men. Additionally, we compared the findings obtained from multiple imputation data with those from complete data (eTable 3, eTable 4, and eTable 5) and confirmed that the results of the analysis in subjects with complete data were similar to the pattern of those in which subjects with missing values were included.

## DISCUSSION

The present study investigated how types of LA were associated with positive SRH among community-dwelling older persons, and whether these associations varied with gender and work status. After both adjustment for potential confounders and mutual adjustment for the other LA, some types of LA were significantly and independently associated with positive SRH: sports activities among older men and non-working women; cooking among older adults without a job; musical activities among working men and non-working women; and technology usage only among working men. Moreover, we found that in non-working women, TV watching was negatively associated with positive SRH.

Numerous studies have reported the favorable health effects of physical activity, while some studies have indicated that above-moderate physical activity is essential for improving health.^19^^,^^31^^,^^32^ In this study, the positive association between LA and positive SRH was strongest in sports activities, while gardening was not associated with positive SRH, regardless of gender or work status. Because sports activities are more likely to include above-moderate physical activity than gardening,^21^ our findings are consistent with the findings of prior studies.^19^^,^^31^^,^^32^ Our results suggest that the level of physical activity plays an important role in older people’s SRH, and this effect depends on neither gender nor work status.

Regarding cooking, a prior study found that community-dwelling older adults who participated in nutrition education-based cooking workshops showed an improvement in dietary habits.^33^ Therefore, older people who cook may have more interest in a healthy diet than those who do not, resulting in behaviors which contribute to a healthier lifestyle. In this study, significant associations between cooking and positive SRH were seen in non-working persons, but not in older workers, regardless of gender. Although we failed to confirm a significant interaction between cooking and work status, older adults without a job may have more time to watch their diet than older workers, leading to a positive association with positive SRH.

Regarding musical activities, we found a positive association with positive SRH in working men and non-working women, a significant interaction effect by work status among men, and effect modification by gender. Possible mechanisms of these results are as follows. First, musical activities need complex and high-level abilities, such as motor skills (eg, playing a musical instrument), memory (eg, singing a song), and the coordination of the auditory, visual, and somatosensory information.^34^ Prior studies reported that musical activities were associated with the prevention of cognitive decline^34^^,^^35^ and the enhancement of emotional well-being^34^^,^^36^ among older adults. Next, the beneficial effects of old-age working may depend on gender and the type of health outcomes.^11^^,^^37^ For example, working in later life had a preventive effect on depression for men,^37^ while preventing IADL decline for women.^11^ Additionally, as shown in Table 2, women were more likely to engage in musical activities than men, and participation in musical activities did not depend on work status. These factors may affect the association between musical activities and positive SRH, leading to different results according to gender and work status.

In this study, men used technology more often for LA than women, and a significant association between technology usage and positive SRH was found only for working men. This finding is partly consistent with a prior study of older adults which reported that men tended to use the Internet more than women, and Internet use was associated with healthy behaviors, such as active exercise, healthy diet, and undergoing a cancer checkup.^27^ Given that technology, such as the Internet and e-mail, is common in the workplace, that the Internet can be a source of health information,^38^ and that men are more engaged in using technology than women, male older workers who engage in technology usage may obtain more health-related information than women and non-working men, thus leading to better health.

Among women, TV watching was negatively associated with positive SRH in non-working persons, and there was a significant interaction between TV watching and work status. Prior studies reported that TV watching time was associated with a higher risk of adverse health outcomes,^24^^,^^39^ and that TV watching time was greater in the older age group than the younger age group.^40^ Additionally, TV watching time was associated positively with leisure-time sedentary behaviors and negatively with leisure-time physical activity in women, but not in men.^41^ Therefore, TV watching may be more strongly linked to a sedentary lifestyle in older women than in older men. Because workforce participation by older women can produce a positive effect on functional capacity^11^ and well-being,^42^ which are the fundamental components of SRH,^15^^,^^17^ female older workers may experience a reduction in the negative effects of TV watching on SRH.

Among working women, there was no association between any type of LA and positive SRH. A prior study of non-institutionalized older adults also reported that after mutual adjustment for LA, significant associations between certain types of LA and survival were observed in men, but not in women.^5^ A possible explanation of our results could be that women represented a small percentage of working people. This may have led to insufficient statistical power, resulting in no associations between different types of LA and positive SRH. Because participation in a greater number of types of LA was associated with an increased prevalence of positive SRH (Table 4), our findings indicate that participation in a variety of LA is important in maintaining the health of female workers.

Our study has several limitations. First, this was a cross-sectional study. Therefore, we could not establish causal relationships. It is highly probable that older people with positive SRH engage actively in LA. Second, SRH, LA, and most of the covariates were based on self-reported data. This might overvalue the association between SRH and LA and cause misclassification.^43^ Third, our study had an inadequate response rate. In particular, we failed to include individuals aged 65–69 years (Table 1). The younger-old might have better health than the older-old. In contrast, because the younger-old are more involved in paid work than the older-old,^44^ they might not have enough time to participate in LA. It is unclear whether this selection bias led us to underestimate or overestimate the SRH/LA association in our results. Fourth, we evaluated the presence or absence of 14 types of LA but failed to assess the frequency of LA. Prior studies of older people suggest that frequency of LA may differ in regards to work status as well as gender,^6^^,^^12^ and that the LA-health relationship may vary with the frequency of LA.^19^^,^^45^ Additional studies based on the frequency of LA are required. Finally, study participants were community-dwelling older persons in an urban area of Japan. Because prior studies indicate regional differences in the level of LA^6^ and the effect of cultural background and national traits on SRH,^16^ we need to be careful in generalizing our findings to older people living in rural areas, metropolitan areas, or other countries apart from Japan.

Despite these limitations, to our knowledge, this is the first study to investigate the association between LA and SRH according to the type of LA, gender, and work status among community-dwelling older people. Our findings demonstrate that not all types of LA are associated with positive SRH, and the relationships between LA and SRH depend on gender and work status as well as the type of LA. In terms of promotion of LA for the purpose of maintaining older adults’ SRH, family physicians and public health nurses should recommend LA, bearing in mind the type of LA most suited to gender and work status.

## APPENDIX A. SUPPLEMENTARY DATA

eAppendix 1. Detailed explanations of covariates and multiple imputations

eTable 1. List of leisure activities

eTable 2. Characteristics of research subjects who responded to the questionnaire

eTable 3. Comparison between the findings obtained from multiple imputation data and those from complete data, based on the mutual adjustment for all 14 types of leisure activities: stratified analyses by work status among men

eTable 4. Comparison between the findings obtained from multiple imputation data and those from complete data, based on the mutual adjustment for all 14 types of leisure activities: stratified analyses by work status among women

eTable 5. Comparison between the findings obtained from multiple imputation data and those from complete data, based on the mutual adjustment for all 14 types of leisure activities: stratified analyses by gender
