# Supplementary material for: Cross-Sectional Association Between Types of Leisure Activities and Self-rated Health According to Gender and Work Status Among Older Japanese Adults
Source: J Epidemiol. 2019 Nov 5;29(11):424–31. doi: 10.2188/jea.JE20180108 (PMC6776474; doi:10.2188/jea.JE20180108)
Supplement: Supplementary file 1 [file je-29-424-s001.pdf]

Supplementary data

eTable 1. List of leisure activities

Type of leisure activities	Examples
Sports activities	Walking, jogging, swimming, golf, tennis, gymnastics, dancing, yoga
Gardening	Home vegetable garden, horticulture, flower-growing, growing of a crop
Musical activities	Playing a music instrument, singing in a choir, singing a Japanese folk song, a Chinese poem, and/or karaoke music
Creative activities	Handicraft, ceramics, painting, photography, home carpentry
Cultural activities	Reading books, learning, composing poems, enjoying flower arrangement, tea ceremony, and calligraphy
Playing games	Playing Go, Japanese chess, and mah-jongg
Sightseeing	Domestic travel, overseas travel, going on a trip by car, visiting shrines, temples, and/or historical sites
Art appreciation	Appreciating music and drama, going to the movie and the theater, looking at pictures and fine art, visiting museums
TV watching	Watching a TV program and a video tape, listening to the radio
Cooking	Making meals and confectionery (a tour of restaurants is not included)
Pet ownership	Owning and looking after a pet
Technology usage	Using information and communication technology (ICT) such as the Internet, e-mails, and mobile/smart phones
Investment	Investing in stocks, bonds, immovables, and/or exchange
Gambling	Horse racing, speedboat racing, pachinko (i.e., Japanese pinball), public lottery

eAppendix 1. Detailed explanations of covariates and multiple imputations

Covariates

Age was categorized as 65–69, 70–74, 75–79, and ≥80 years. Marital status was categorized as either currently married or not married. Education was categorized as high (junior college or higher), middle (high school), and low (junior high school). Subjective economic status was categorized as rich (very rich/rich), fair, and poor (poor/very poor). BMI (kg/m^2) was categorized as normal (18.5 to <25.0), underweight (<18.5), and overweight (≥25.0). Information on chronic diseases included hypertension, hyperlipidemia, diabetes mellitus, cardiovascular disease, cerebrovascular diseases, and cancer. The number of chronic diseases was categorized as zero, one, and ≥two. Alcohol intake was categorized as daily/occasional drinker and non/social drinker. Smoking history was categorized as ex/current smoker and never-smoker. Waking time per day was categorized as ≥1 hour and <1 hour. Depression was measured with the five-item short form of the Geriatric Depression Scale.[1] Cognitive functioning was measured with the Cognitive Performance Scale.[2]

Multiple imputations

Using logistic regression, we created five sets of imputation data and performed

analyses on the complete pooled data set. The independent variable, outcome, gender, work status, and all the covariate variables were entered into the imputation procedure. For this study, there were no missing data on gender and age. Marital status (0.3% missing), education (0.8% missing), subjective economic status (0.2% missing), BMI (2.1% missing), chronic diseases (2.2% missing), alcohol intake (0.4% missing), smoking (0.8% missing), walking time (1.8% missing), depression (0.5% missing), and cognitive functioning (1.7% missing) were imputed as ordinal variables.

REFERENCES

1. Hoyl MT, Alessi CA, Harker JO, et al. Development and testing of a five-item version of the Geriatric Depression Scale. J Am Geriatr Soc. 1999;47:873–878.
2. Morris JN, Fries BE, Mehr DR, et al. MDS Cognitive Performance Scale. J Gerontol. 1994;49:M174–182.

eTable 2. Characteristics of research subjects who responded to the questionnaire

	Men (n=4,044)		Women (n=4,617)	
	Working (n=1,208) n (%)	Not working (n=2,836) n (%)	Working (n=687) n (%)	Not working (n=3,930) n (%)
**Socio-demographic factors**				
Age, years				
65–69	616 (51.0)	664 (23.4)	405 (59.0)	1,174 (29.9)
70–74	343 (28.4)	816 (28.8)	177 (25.8)	1,082 (27.5)
75–79	162 (13.4)	755 (26.6)	77 (11.2)	848 (21.6)
≥80	87 (7.2)	601 (21.2)	28 (4.1)	826 (21.0)
Marital status				
Currently married	1,105 (91.5)	2,494 (87.9)	443 (64.5)	2,460 (62.6)
Not married	102 (8.4)	336 (11.8)	238 (34.6)	1,459 (37.1)
Missing	1 (0.1)	6 (0.2)	6 (0.9)	11 (0.3)
Education				
High: junior college or higher	481 (39.8)	835 (29.4)	186 (27.1)	768 (19.5)
Middle: high school	511 (42.3)	1,327 (46.8)	370 (53.9)	2,155 (54.8)
Low: junior high school or below	209 (17.3)	662 (23.3)	127 (18.5)	964 (24.5)
Missing	7 (0.6)	12 (0.4)	4 (0.6)	43 (1.1)
Subjective economic status				
Rich: very rich/rich	373 (30.9)	589 (20.8)	194 (28.2)	923 (23.5)
Middle: fair	650 (53.8)	1,623 (57.2)	346 (50.4)	2,271 (57.8)
Poor: poor/very poor	185 (15.3)	622 (21.9)	145 (21.1)	724 (18.4)
Missing	0 (0.0)	2 (0.1)	2 (0.3)	12 (0.3)
**Health status**				
Body mass index (kg/m^2)				
Normal (18.5–24.9)	856 (70.9)	2,036 (71.8)	505 (73.5)	2,687 (68.4)
Underweight (<18.5)	46 (3.8)	122 (4.3)	53 (7.7)	384 (9.8)
Overweight (≥25.0)	278 (23.0)	621 (21.9)	123 (17.9)	765 (19.5)
Missing	28 (2.3)	57 (2.0)	6 (0.9)	94 (2.4)
Number of chronic diseases				
None	434 (35.9)	874 (30.8)	331 (48.2)	1,539 (39.2)
One	509 (42.1)	1,212 (42.7)	243 (35.4)	1,586 (40.4)
≥Two	261 (21.6)	706 (24.9)	88 (12.8)	691 (17.6)
Missing	4 (0.3)	44 (1.6)	25 (3.6)	114 (2.9)

eTable 2. Continued

	Men (n=4,044)		Women (n=4,617)	
	Working (n=1,208) n (%)	Not working (n=2,836) n (%)	Working (n=687) n (%)	Not working (n=3,930) n (%)
**Health behaviors**				
Alcohol intake				
Daily/occasional drinkers	831 (68.8)	1,711 (60.3)	248 (36.1)	1,047 (26.6)
Non/social drinkers	374 (31.0)	1,116 (39.4)	437 (63.6)	2,860 (72.8)
Missing	3 (0.2)	9 (0.3)	2 (0.3)	23 (0.6)
Smoking history				
Ex/current smokers	886 (73.3)	2,074 (73.1)	91 (13.2)	344 (8.8)
Never-smokers	317 (26.2)	746 (26.3)	593 (86.3)	3,540 (90.1)
Missing	5 (0.4)	16 (0.6)	3 (0.4)	46 (1.2)
Walking time per day				
1 hour or more	481 (39.8)	906 (31.9)	273 (39.7)	1,227 (31.2)
Less than 1 hour	718 (59.4)	1,881 (66.3)	400 (58.2)	2,615 (66.5)
Missing	9 (0.7)	49 (1.7)	14 (2.0)	88 (2.2)
**Mental function**				
Depression measured by the five-item version of the Geriatric Depression Scale (score range 0–5)				
No depression (0–1)	1,047 (86.7)	2,056 (72.5)	559 (81.4)	2,826 (71.9)
Depression (2 or more)	156 (12.9)	769 (27.1)	126 (18.3)	1,077 (27.4)
Missing	5 (0.4)	11 (0.4)	2 (0.3)	27 (0.7)
Cognitive functioning measured by the Cognitive Performance Scale (score range 0-6)				
Intact (0)	1,045 (86.5)	2,192 (77.3)	620 (90.2)	3,197 (81.3)
Poor (1 or more)	153 (12.7)	606 (21.4)	51 (7.4)	649 (16.5)
Missing	10 (0.8)	38 (1.3)	16 (2.3)	84 (2.1)
**Outcome indicator**				
Self-rated health measured by one item of the eight-item Short-Form Health Survey (SF-8)				
Excellent	38 (3.1)	65 (2.3)	18 (2.6)	73 (1.9)
Very good	255 (21.1)	463 (16.3)	150 (21.8)	510 (13.0)
Good	712 (58.9)	1,580 (55.7)	398 (57.9)	2,187 (55.6)
Fair	168 (13.9)	582 (20.5)	99 (14.4)	914 (23.3)
Poor	29 (2.4)	106 (3.7)	16 (2.3)	173 (4.4)
Very poor	6 (0.5)	40 (1.4)	6 (0.9)	73 (1.9)

eTable 3. Comparison between the findings obtained from multiple imputation data and those from complete data, based on the mutual adjustment for all 14 types of leisure activities: stratified analyses by work status among men

Type of leisure activities	Working men		Non-working men		All men	
	MI data n=1,208	Complete data n=1,154	MI data n=2,836	Complete data n=2,638	MI data n=4,044	Complete data n=3,792
	OR[a] (95% CI)	OR[a] (95% CI)	OR[a] (95% CI)	OR[a] (95% CI)	*P* for interaction[b]	*P* for interaction[b]
Sports activities	**1.46 (1.07-2.00)**	**1.55 (1.12-2.14)**	**1.33 (1.04-1.69)**	1.27 (0.99-1.63)	0.555	0.323
Gardening	0.97 (0.73-1.31)	1.04 (0.77-1.40)	0.99 (0.80-1.22)	0.97 (0.78-1.20)	0.996	0.722
Musical activities	**1.44 (1.01-2.05)**	**1.50 (1.04-2.15)**	0.96 (0.74-1.24)	0.96 (0.73-1.25)	**0.046**	**0.038**
Creative activities	1.19 (0.77-1.83)	1.12 (0.72-1.75)	1.20 (0.91-1.58)	1.25 (0.94-1.66)	0.683	0.997
Cultural activities	1.09 (0.74-1.60)	1.10 (0.74-1.64)	0.88 (0.68-1.14)	0.90 (0.69-1.17)	0.207	0.275
Playing games	1.01 (0.70-1.45)	1.01 (0.70-1.46)	0.85 (0.66-1.09)	0.87 (0.67-1.12)	0.202	0.218
Sightseeing	1.00 (0.72-1.39)	0.93 (0.67-1.30)	1.18 (0.93-1.50)	1.19 (0.93-1.52)	0.910	0.671
Art appreciation	1.20 (0.85-1.71)	1.18 (0.82-1.70)	0.99 (0.77-1.27)	0.99 (0.76-1.28)	0.251	0.351
TV watching	0.82 (0.59-1.13)	0.84 (0.60-1.17)	0.87 (0.69-1.10)	0.86 (0.67-1.10)	0.714	0.618
Cooking	0.89 (0.52-1.54)	0.89 (0.51-1.56)	**1.65 (1.18-2.33)**	**1.67 (1.17-2.37)**	0.149	0.148
Pet ownership	0.85 (0.58-1.27)	0.82 (0.54-1.22)	0.87 (0.64-1.18)	0.89 (0.66-1.22)	0.914	0.705
Technology usage	**1.41 (1.01-1.96)**	**1.53 (1.09-2.15)**	1.08 (0.86-1.37)	1.13 (0.89-1.44)	0.189	0.181
Investment	0.88 (0.61-1.29)	0.82 (0.56-1.21)	1.00 (0.77-1.31)	0.96 (0.73-1.27)	0.968	0.989
Gambling	0.79 (0.54-1.15)	0.80 (0.54-1.17)	0.86 (0.65-1.13)	0.84 (0.63-1.13)	0.756	0.851

CI, confidence interval; MI, multiple imputation; OR, odds ratio.

Non-participation in each activity was set as the reference group. Results in bold indicate P <0.05.

[a]Adjusted for covariates, mental function, and all 14 leisure activity variables.

[b]P for interaction between work status and each type of leisure activities.

eTable 4. Comparison between the findings obtained from multiple imputation data and those from complete data, based on the mutual adjustment for all 14 types of leisure activities: stratified analyses by work status among women

Type of leisure activities	Working women		Non-working women		All women	
	MI data n=687	Complete data n=616	MI data n=3,930	Complete data n=3,522	MI data n=4,617	Complete data n=4,138
	OR[a] (95% CI)	OR[a] (95% CI)	OR[a] (95% CI)	OR[a] (95% CI)	*P* for interaction[b]	*P* for interaction[b]
Sports activities	1.19 (0.79-1.78)	1.09 (0.70-1.68)	**1.74 (1.41-2.15)**	**1.71 (1.37-2.14)**	0.312	0.238
Gardening	1.32 (0.86-2.01)	1.31 (0.83-2.06)	1.06 (0.87-1.29)	1.06 (0.86-1.31)	0.298	0.328
Musical activities	1.07 (0.69-1.66)	1.02 (0.64-1.64)	**1.59 (1.29-1.95)**	**1.65 (1.33-2.05)**	0.175	0.111
Creative activities	0.82 (0.52-1.30)	0.89 (0.54-1.46)	0.92 (0.74-1.15)	0.94 (0.75-1.18)	0.962	0.933
Cultural activities	0.98 (0.62-1.53)	0.99 (0.61-1.61)	0.93 (0.75-1.16)	0.96 (0.76-1.20)	0.562	0.701
Playing games	2.10 (0.67-6.62)	2.22 (0.61-8.07)	0.82 (0.53-1.28)	0.82 (0.51-1.30)	0.304	0.276
Sightseeing	1.19 (0.75-1.89)	1.33 (0.81-2.18)	1.03 (0.83-1.29)	0.99 (0.78-1.25)	0.338	0.235
Art appreciation	1.22 (0.78-1.92)	1.09 (0.68-1.77)	1.03 (0.82-1.29)	1.07 (0.84-1.35)	0.366	0.718
TV watching	1.10 (0.72-1.67)	1.06 (0.67-1.67)	**0.69 (0.56-0.85)**	**0.71 (0.57-0.88)**	**0.043**	0.084
Cooking	1.37 (0.89-2.12)	1.40 (0.88-2.25)	**1.28 (1.03-1.60)**	1.22 (0.97-1.54)	0.566	0.491
Pet ownership	0.68 (0.39-1.18)	0.53 (0.28-1.01)	1.10 (0.84-1.45)	1.09 (0.82-1.44)	0.198	0.110
Technology usage	0.95 (0.60-1.50)	0.96 (0.58-1.57)	1.13 (0.88-1.44)	1.16 (0.90-1.50)	0.802	0.904
Investment	1.33 (0.73-2.42)	1.65 (0.86-3.16)	1.07 (0.78-1.47)	0.99 (0.70-1.39)	0.527	0.237
Gambling	1.01 (0.46-2.19)	0.94 (0.41-2.13)	0.70 (0.42-1.16)	0.72 (0.43-1.22)	0.338	0.429

CI, confidence interval; MI, multiple imputation; OR, odds ratio.

Non-participation in each activity was set as the reference group. Results in bold indicate *P* <0.05.

[a]Adjusted for covariates, mental function, and all 14 leisure activity variables.

[b]*P* for interaction between work status and each type of leisure activities.

eTable 5. Comparison between the findings obtained from multiple imputation data and those from complete data, based on the mutual adjustment for all 14 types of leisure activities: stratified analyses by gender

Type of leisure activities	Men		Women		All	
	MI data n=4,044	Complete data n=3,792	MI data n=4,617	Complete data n=4,138	MI data n=8,661	Complete data n=7,930
	OR[a] (95% CI)	OR[a] (95% CI)	OR[a] (95% CI)	OR[a] (95% CI)	*P* for interaction[b]	*P* for interaction[b]
Sports activities	**1.38 (1.15-1.67)**	**1.37 (1.13-1.67)**	**1.60 (1.33-1.92)**	**1.55 (1.28-1.88)**	0.416	0.471
Gardening	0.99 (0.84-1.17)	1.00 (0.84-1.19)	1.10 (0.92-1.32)	1.10 (0.91-1.33)	0.534	0.588
Musical activities	1.08 (0.88-1.32)	1.09 (0.88-1.34)	**1.47 (1.23-1.77)**	**1.52 (1.25-1.84)**	**0.022**	**0.022**
Creative activities	1.19 (0.95-1.50)	1.21 (0.95-1.53)	0.90 (0.74-1.09)	0.93 (0.76-1.14)	0.066	0.095
Cultural activities	0.93 (0.76-1.16)	0.95 (0.77-1.19)	0.94 (0.78-1.14)	0.96 (0.79-1.18)	0.847	0.812
Playing games	0.91 (0.75-1.12)	0.93 (0.76-1.15)	0.89 (0.60-1.35)	0.89 (0.58-1.38)	0.935	0.948
Sightseeing	1.12 (0.93-1.36)	1.10 (0.90-1.34)	1.06 (0.87-1.30)	1.05 (0.85-1.29)	0.889	0.962
Art appreciation	1.04 (0.85-1.27)	1.04 (0.84-1.28)	1.06 (0.87-1.30)	1.06 (0.86-1.31)	0.913	0.980
TV watching	0.85 (0.71-1.03)	0.86 (0.70-1.04)	**0.76 (0.63-0.91)**	**0.77 (0.64-0.94)**	0.474	0.533
Cooking	**1.40 (1.05-1.86)**	**1.40 (1.04-1.89)**	**1.28 (1.05-1.55)**	1.23 (1.00-1.50)	0.689	0.558
Pet ownership	0.89 (0.70-1.12)	0.89 (0.70-1.14)	0.99 (0.77-1.26)	0.95 (0.74-1.23)	0.621	0.804
Technology usage	1.20 (0.99-1.45)	**1.26 (1.04-1.53)**	1.11 (0.90-1.38)	1.14 (0.91-1.42)	0.539	0.511
Investment	0.97 (0.78-1.20)	0.92 (0.73-1.14)	1.10 (0.84-1.46)	1.07 (0.80-1.44)	0.390	0.377
Gambling	0.84 (0.67-1.05)	0.83 (0.66-1.05)	0.78 (0.52-1.19)	0.79 (0.51-1.21)	0.932	0.989

CI, confidence interval; MI, multiple imputation; OR, odds ratio.

Non-participation in each activity was set as the reference group. Results in bold indicate *P* <0.05.

[a]Adjusted for covariates, mental function, work status, and all 14 leisure activity variables

[b]*P* for interaction between gender and each type of leisure activities.
